# Effectiveness of the Contours Vibes™ soothing vibrations infant and toddler crib mattress on infant and maternal sleep quality and health outcomes: an intervention study

**DOI:** 10.5935/1984-0063.20210014

**Published:** 2022

**Authors:** Heather Hausenblas, Stephanie Hooper

**Affiliations:** Jacksonville University, School of Applied Health Sciences - Jacksonville - FL - United States.

**Keywords:** Health, Infant, Sleep

## Abstract

**Objectives:**

The purpose was to examine the effectiveness of the Contours Vibes™ Crib Mattress on infants’ and maternal sleep quality and health.

**Material and Methods:**

Participants were 24 mothers and their infants. For Week 1 the infants slept on their current mattress (Baseline). For Week 2 they slept on the Vibrating Mattress without using the vibrating feature (VMC). For Week 3 they slept on the Vibrating Mattress using the vibrating feature (VMA). Sleep quality and health assessments were completed at Baseline and following VMA and VMC conditions.

**Results:**

Sleep quality and health outcomes improved significantly for VMA compared to Baseline and VMC. VMC had significant improvements in some sleep and health outcomes compared to Baseline. No adverse events were reported.

**Conclusion:**

The mattress is a simple, noninvasive, and non-pharmacological intervention that improved infant and maternal sleep quality and health. Further research is need examining the longitudinal effects of the mattress.

## INTRODUCTION

Infant sleep issues such as bedtime/naptime problems and night waking are common, with a prevalence of about 30%^1,2^. The most common types of sleep problems include difficulties initiating sleep and maintaining sleep, and they often cause significant parental and infant distress^2^.

For example, infant sleep problems are associated with adverse consequences for mothers including fatigue^3^, postnatal depression, and poorer physical and mental health^4^. Sleep issues are also associated with both acute and chronic physical, behavioral, and emotional problems for the infant^5,6^. Thus, early science-based interventions are needed to prevent and treat infant sleep issues for the health and well-being of both the mother and infant^7^.

A systematic review found short-term positive infant sleep effects of the behavioral strategies of establishing consistent routines and controlled crying; but inconsistent evidence for education interventions^8^. Although empirical studies support the potential for some behavioral interventions, parental education and intervention adherence are barriers to successful implementation. Thus, it is necessary to identify alternative interventions with high ease of implementation, tolerability, adherence, and effectiveness for improving infant sleep.

Vibrating technology (e.g., repetitive and rhythmic motions) is a technological approach that may enhance the infant sleep experience. Relative to behavioral interventions, vibrating technology has several advantages including ease of use, adherence, and implementation. The limited research supports that the low-frequency vibration of a running train makes people fall asleep^9^ and anecdotal support for vibrating infant chairs and swings exists. Research is needed, however, to examine the effectiveness of vibrating mattresses to improve sleep quality and well-being of both infants and parents^10^.

This study addresses this void by examining the effectiveness of a novel vibrating mattress to improve infants sleep quality. The Contours Vibes™ crib mattress is designed to help soothe and comfort infants and toddlers by offering a gentle vibration throughout the mattress. The primary study purpose was to examine the effectiveness of this vibrating mattress technology on infants’ and maternal sleep quality. The secondary purpose was to examine the mattresses effects on maternal and infant psychosocial outcomes. We hypothesized that when the infants slept on the vibrating mattress significant improvements in both parental and infant sleep quality and psychosocial outcomes (e.g., perceived stress, health-related quality of life) would be evidenced.

## MATERIAL AND METHODS

### Participants

Participants were 26 mothers (M age=33.48, SD=6.57) and their infants who had moderately high fussiness (M age=8.56 months, SD=2.99; n=13 male and n=13 female). Participants were from the U.S.A. and were from 18 different states. Inclusion criteria were: (1) infants 3 to 12 months of age, (2) infants sleep in a full-size crib, (3) parental perceptions of infant sleep issues (i.e., scoring 6 or greater on the infant sleep fussiness item), (4) infants slept in their current environment for at least 3 weeks, and (5) parent speaks English. The mothers indicated that their infant fussiness was M=8.04 (SD=1.10, range=6 to 10) on a 10 point fussiness scale where 1 = not fussy/no sleep issues and 10 = severe/fussy sleeper.

### Procedures and design

**Figure 1.**
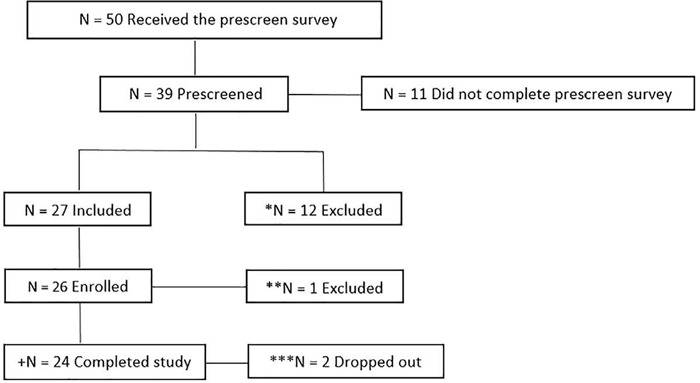
Flowchart of the participant recruitment. Notes: *N = 12 excluded for not meeting inclusion criteria; **N = 1 excluded because participant stopped responding after completing the prescreen; ***N = 2 dropped out due to person issues unrelated to the study; +N = 1 excluded from analyses due to language barrier leading to inability to complete surveys accurately.

Interested women (n=901) from a Kolcraft listserve who met our inclusion criteria (n=492) were randomly contacted to determine eligibility (n=50; see Figure 1 for the participant flowchart).

Participants who met our inclusion criteria and signed an informed consent where enrolled in the study (n=26). For week 1 of the study, the infants slept on their current mattress (baseline). For week 2 the infants slept on the vibrating mattress without using the vibrating feature (vibrating mattress control - VMC). For week 3 the infants slept on the vibrating mattress using the vibrating feature (vibrating mattress active - VMA).

This vibrating mattress contours is a new category for the Kolcraft brand, Chicago, IL, and is available for purchase in 2021 (www.contoursbaby.com). The mattress is a breathable airflow pocketed and posture supported design with 201 resilient springs. The waterproof mattress cover is cotton with PVC free barrier. The two-stage design has a firmer infant side and a softer toddler side. The mattress is battery operated and has three vibration mode settings of “gentle”, “heartbeat”, and “whisper”. The vibration mechanism is a CPU controlled, battery powered micro-motor. When the unit is set to the heartbeat mode a rhythmic pattern vibrates mimicking mother’s heartbeat, in whisper mode it changes to a soft constant vibration, and then in gentle mode the intensity increases slightly in a constant vibration. The mode was self-selected by the mothers. The vibrations are constant pulsations that were either turned off either manually by the mother or by using a timer within the mattress.

The mothers received a daily reminder regarding which week they were in (i.e., baseline, VMC, or VMA) and to complete a daily diary assessing parental and infant sleep quality, mood, and adherence. They also completed self-report assessments of their mood, daytime fatigue, anxiety, perceived stress, productivity, infants’ sleep quality, colic symptoms, and health-related quality of life at baseline and following each of the mattress conditions. Participants maintained their current lifestyle behaviors for the study duration. The participants kept the mattress following the completion of the study and they were also provided monetary compensation for participating.

A total of 26 adults and infants were enrolled in the study and 24 completed the study, representing an adherence rate of 92%. Two participants dropped out due to personal reasons unrelated to the study. Data were collected electronically via secured links.

### Measures

**Pittsburgh Sleep Quality Index:** The Pittsburgh Sleep Quality Index is a well-known, validated, and reliable instrument used to measure sleep quality such as perceived sleep quality, sleep latency (how long it takes to fall asleep); sleep duration; habitual sleep efficiency (how long a person is asleep in comparison to their time in bed); sleep disturbances (i.e., noise, temperature, pain, nocturia), and daytime dysfunction (sleepiness, concentration)^11^. Higher scores indicate poorer sleep quality on a continuous scale and a score of 5 or greater indicates poor sleep quality.

**Profile Of Mood States (POMS):** The POMS is a validated research tool designed to measure the mood states of tension, anger, vigor, fatigue, depression, and confusion. The total mood disturbance score is computed by summing the tension, depression, anxiety, fatigue, and confusion scores with the vigor scores subtracted. Higher scores indicate increased mood disturbance^12^.

**Flinders Fatigue Scale:** The Flinders Fatigue Scale Must Be measures characteristics of daytime fatigue (e.g., frequency and severity). The items tap into commonly reported themes of how problematic fatigue is, the consequences of daytime fatigue, and perception of fatigue’s association with sleep, with higher scores indicating greater fatigue. A description of the term “fatigue” is provided in the initial instructions to the scale of: “ We are interested in the extent that you have felt fatigued (tired, weary, or exhausted) over the last two weeks. We do not mean feelings of sleepiness (the likelihood of falling asleep)”. This scale is a validated has good to excellent psychometric properties^13^.

**Trait Anxiety Inventory:** The trait anxiety inventory assesses levels of anxiety (e.g., tense, calm, or upset) experienced over the last two weeks. This inventory is a validated for research, with higher scores indicating higher anxiety levels^11^.

**Perceived Stress Scale:** The shortened version of the Perceived Stress Scale must be (4-items) measures the degree to which situations are appraised as stressful. That is, “How unpredictable, uncontrollable, and overloaded respondents find their lives” during the past two weeks. This scale is a validated research inventory and higher scores indicate higher perceived stress^12^.

**Infant Health-related Quality of Life scale:** The infant health-related quality of life scale assesses the following health items of sleeping, feeding, breathing, stooling/poo, mood, skin, and interaction, each relevant up to 1 year of age. This scale has good to excellent psychometric properties and a higher score indicates poorer infant health^13^.

**Brief Infant Colic Questionnaire:** The brief infant colic questionnaire is a validated research inventory that assesses colic symptoms such as nocturnal and daytime sleep duration; number of night wakening’s; duration of wakefulness during the night hours; nocturnal sleep-onset time (the time when the child falls asleep for the night); settling time (latency to falling asleep for the night); and method of falling asleep. Each item is assessed individually to determine the specific level of colic.

**Infant Sleep Quality Questionnaire:** The infant sleep quality questionnaire is a validated research tool that assesses nap and sleep quality (e.g., nocturnal sleep duration, night wakings, method of falling asleep. Each item is assessed individually to determine infant sleep quality.

**Daily Diary:** The daily diary assessed adherence, mattress useability/likability, and infant and parental sleep quality and mood.

### Data analysis

Data were analyzed for normality using Shapiro-Wilk Test and the skewness and kurtosis values of the items. Descriptive statistics were expressed in mean (SD) and percentage/count formats. Repeated measures ANOVAs were used to examine time differences (*p*’s=.05). Data were analyzed using Excel and SPSS (Version 24).

## RESULTS

Findings from the Infant Sleep Questionnaire. revealed that VMA resulted in significant improvements in infant nap duration, nap quality, nighttime sleep, fussiness, and sleep issues, and maternal sleep quality compared to both Baseline and VMC, *p*’s<.05. Also, VMC resulted in significant improvements in infant nap duration, nap quality, nighttime sleep, fussiness, sleep issues, and maternal sleep quality compared to baseline, *p*’s<. 05 (see Table 1).

**Table 1.** Infant sleep quality and infant health-related quality of life descriptive and ANOVA statistics across conditions.

Questionnaire items	Baseline M (SD)	VM C M (SD)	VMA M (SD)	ANOVA df (2,44)
Infant sleep quality scale				
Amount of naps^†^	1.74 (0.62)	1.48 (0.73)	1.52 (0.67)	F= 4.46, p = .02
Length of naps^*#^	48.30 (24.16)	48.78 (24.33)	59.83 (27.43)	F = 3.29, p = .05
Good napper (Scale 1 to 10) *#	4.30 (2.24)	5.09 (2.41)	6.09 (2.94)	F= 5.97, p = .005
Good nighttime sleeper (Scale 1 to 10) ^*#^	4.04 (2.1)	4.52 (1.93)	6.22 (2.73)	F = 12.08, p < .001
Fussiness of infant sleep (Scale 1 to 10) ^*#^	7.30 (1.72)	6.87 (1.96)	5.26 (2.58)	F = 9.61, p < .001
Severity of infant sleep issues (Scale 1 to 10) *^#†^	7.87 (0.97)	6.70 (1.52)	4.61 (2.25)	F = 43.69, p < .001
Mother sleep quality (Scale 1 to 10)^*#^	4.52 (1.95)	5.39 (1.78)	7.04 (2.16)	F = 12.28, p < .001
Infant health-related quality of life				
Sleep quality*^#†^	3.17 (0.65)	2.83 (0.78)	2.09 (1.12)	F = 15.22, p < .001
Feeding	1.26 (0.62)	1.35 (0.65)	1.22 (0.60)	F = 0.34, p = 0.71
Breathing	1.04 (0.21)	1.13 (0.46)	1.04 (0.21)	F = 0.66, p = .52
Stool	1.35 (0.78)	1.43 (0.84)	1.17 (0.49)	F = 2.27, p = 0.11
Mood^*#^	1.65 (0.49)	1.61 (0.58)	1.26 (0.45)	F = 7.11, p = 0.002
Skin^*^	1.35 (0.65)	1.22 (0.6)	1.09 (0.42)	F = 2.39, p = .104
Interaction^*#^	1.74 (0.45)	1.52 (0.51)	1.30 (0.47)	F = 8.21, p < .001
Total Score^*#^	11.57 (1.90)	11.09 (1.93)	9.17 (1.99)	F = 18.62, p < .001

**Notes:** ^*^ = Significant differences from BL to VMA; ^#^ = Significant differences from VMC to VMA, ^†^ = Significant differences from BL to VMC.

For the Infant Health-Related Quality of life significant improvements in sleep quality, mood, interaction, and overall quality of life was evidenced for VMA compared to baseline and VMC, *p*’s<.05. Skin health also improved significantly from VMA to baseline, *p*<.05. Sleep quality improved significantly from Baseline to VMC, *p*<.05 (see Table 1).

The daily diary revealed significant improvements in Infant and maternal sleep duration, infant fussiness, infant length of time to fall asleep, and maternal mood for the VMA compared to baseline (see Table 2). For the Brief Infant Colic Questionnaire, improvements in colic symptoms of eating behaviors, being able to go to sleep by himself/herself, crankiness level, and happiness level were evidenced for the VMA compared to both baseline and VMC, *p*’s<.05.

**Table 2.** Descriptive and ANOVA statistics for the daily diary by condition.

Item	Baseline M (SD)	VMC M (SD)	VMA M (SD)	ANOVA df (2, 268)
Length of sleep at night for infant	7.53 (2.11)	8.03 (2.17)	8.68 (1.57)	^*#^†F=14.41, p<.001
Length of time it took infant to fall asleep at night	1.97 (3.21)	1.42 (2.69)	1.14 (2.5)	^*^F=18.76, p=.01
How many times infant woke during the night	3.22 (2.01)	2.67 (2.06)	2.27 (1.87)	^*#^F=6.10, p=0.003
How many times infant napped	1.57 (0.91)	1.52 (0.84)	1.49 (0.95)	F=0.04, p=.96
How much sleep mother got	5.51 (1.66)	6.03 (1.72)	6.6 (1.56)	^*#^†F = 15.41, p<.001
Fussiness of infant sleep (1 = No fussiness and 10 = Severe fussiness)	6.81 (2.2)	6.07 (2.22)	4.89 (2.55)	^*#^†F=23.05, *p*<.001
Mother’s mood (1 = Negative mood and 10 = Positive mood)	6.1 (1.94)	6.79 (1.99)	7.57 (1.72)	^*#†^F(2,268)=18.76, *p*<0.001

Notes: ^*^ = Significant differences from BL to VMA; ^#^ = Significant differences from VMC to VMA, ^†^ = Significant differences from BL to VMC.

Finally, findings revealed significant improvements for parental sleep quality (i.e., Pittsburgh Sleep Quality Index), mood (Profile of Mood States), anxiety (i.e., trait anxiety inventory), perceived stress (i.e., perceived stress scale), and daytime fatigue (i.e., Flinder’s fatigue scale) for VMA compared to baseline and VMC, *p*’s<.05. Mothers also reported significantly less fatigue for VMC compared to baseline, *p*<.05 (see Table 3). Finally, based on the PSQI poor sleep quality category, 87% (n=20) of the mothers were categorized as poor sleep quality during the control and VMC conditions compared to 52% (n=12) in the VMA condition, *X^2^*(1), *p*=0.01.

**Table 3.** Descriptive and ANOVA information for parental sleep quality, mood, anxiety, perceived stress, and fatigue.

Item	Baseline M (SD)	VMC M (SD)	VMA M (SD)	ANOVA df(2,44)
Pittsburg Sleep Quality Index	8.87 (3.86)	8.3 (3.53)	5.7 (3.66)	^*#^F=12.05, p<.001
Profile Of Mood States	160.3 (18.59)	153.65 (18.69)	147.57 (15.57)	^*#^F=6.86, p=.003
Anxiety Inventory	47.97 (12.38)	44.93 (12.51)	37.68 (14.58)	^*#^F=8.03, p=.001
Perceived Stress Scale	6.00 (3.09)	5.26 (2.83)	4.00 (3.67)	^*#^F=6.33, p=.004
Flinder’s Fatigue Scale	18.96 (7.66)	15.52 (6.42)	11.13 (7.43)	^*#^†F=15.68, p<.001

**Notes:** ^*^ = Significant differences from BL to VMA; ^#^ = Significant differences from VMC to VMA, ^†^ = Significant differences from BL to VMC.

No adverse events were reported and 96% of the mothers indicated they would continue to have their infant sleep on the vibrating mattress. The most frequency selected vibration mode was whisper (35%), followed by gentle (32%), heartbeat (20%), and no preference (13%).

## DISCUSSION

The Contours Vibes™ crib mattress is designed to help soothe and comfort infants and toddlers by offering a gentle vibration throughout the mattress. The study purpose was to examine the effectiveness of this vibrating mattress technology on infants’ and maternal sleep quality and health outcomes. In support of our hypothesis, we found that the VMA resulted in significant improvements in sleep quality and health outcomes for both the infant and mother. A brief discussion of the findings and future research directions are discussed below.

First, both infant and maternal sleep quality improved significantly when the vibrating mattress feature was used. The VMA resulted in significant improvements in infant nap duration, nap quality, nighttime sleep, fussiness, and sleep infant quantity. To a lesser extent, VMC resulted in significant improvements in infant nap duration, nap quality, nighttime sleep, fussiness, sleep issues, and maternal sleep quality compared to baseline.

For infant health-related quality of life significant improvements in sleep quality, mood, interaction, and overall quality of life was evidenced for the VMA compared to baseline and VMC. Skin health also improved significantly from VMA to baseline. Sleep quality improved significantly from baseline to VMC. For colic symptoms significant improvements were evidenced for eating behaviors, being able to go to sleep by himself/herself, crankiness level, and happiness level were evidenced for the VMA compared to baseline and VMC. Finally, we found significant improvements for maternal mood, anxiety, perceived stress, and daytime fatigue while using VMA compared to baseline and VMC. Mothers also reported significantly less fatigue for VMC compared to baseline.

Poor sleep quality is associated with increases in signs of intrinsic skin aging, poor skin barrier repair, and reduced appearance quality in adults^14^. No located research on skin health and sleep quality in infants was found. Thus, further research is needed examining the effects of sleep quality of infants skin health.

Finally, based on the PSQI poor sleep quality category, a significant improvement in poor sleep quality was found from the baseline/VMC to VMA condition. More specifically, 87% of the mothers during the baseline and VMC were categorized as poor sleep quality compared to 52% in the VMA condition. A meta-analytic review found that 67.2% of postpartum women reported poor sleep quality (based on their PSQI scores)^15^. Our study findings add to the extant literature that poor sleep quality is common in postpartum women. Given the negative impact of poor sleep quality on health outcomes and well-being, interventions, such as the vibrating mattress, are needed to improve sleep quality in this special population.

Further research should examine the effects of the vibrating mattress on postpartum depression because poor sleep quality is a risk factor for this mental health disorder^16^. Research is needed examining how the vibrating mattress longitudinally impacts the sleep and health of infants and both parents using objective and self-report assessments in a variety of environments (e.g., hospitals). Research examining the moderating effect and preferences of the vibration mode on sleep quality is recommended. In summary, the mattress was well-tolerated and may be a simple, non-invasive, and non-pharmacological intervention to promote improve both infant and maternal sleep quality/quantity, colic symptoms, health-related quality of life, mood, anxiety, fatigue, and stress.
